# Vitamins A and D and Their Combinations for Breast and Colorectal Cancers: Analysis of the Clinical, Epidemiological, Preclinical and Transcriptomic Data

**DOI:** 10.3390/ph18111684

**Published:** 2025-11-06

**Authors:** Temitope O. Lawal, Bolanle A. Adeniyi, Gail B. Mahady

**Affiliations:** 1 Department of Pharmacy Practice, Retzky College of Pharmacy, University of Illinois, Chicago, IL 60612, USA; lawaltemitope8@gmail.com (T.O.L.); bolaadeniyi06@gmail.com (B.A.A.); 2 Department of Pharmacy Practice, Retzky College of Pharmacy, World Health Organization Collaborating Center for Traditional Medicine, University of Illinois, Chicago, IL 60612, USA

**Keywords:** breast cancer, cholecalciferol, calcifediol, calcitriol, colorectal cancer, retinoids, vitamins A and D

## Abstract

**Background and Objectives**: Vitamins A and D have been reported to improve cancer outcomes. In this work, we reviewed recent meta-analyses, preclinical, and transcriptomics data for these vitamins and combinations for breast and colon cancers. **Methods**: Searches for meta-analyses, preclinical, and transcriptomic data for vitamins A and D in breast and colorectal cancers were conducted using electronic databases from June 2012 to May 2025. Studies describing the effects of vitamin A and D levels (through diet, supplementation, and serum concentrations) on the risk, prognosis, metastasis, and survival rates of breast and colon cancer patients, and the doses needed to achieve these endpoints, were included. Preclinical and transcriptomics studies investigating combinations of vitamins A and D were also reviewed. **Results**: The reviewed studies showed an inverse correlation between vitamin A intake and the risk and survival rates of breast cancers. Sufficient vitamin D3 levels were associated with improved survival outcomes, lower tumor grades, and less ER- or triple-negative breast cancers. For colorectal cancers, meta-analyses showed conflicting results for vitamin A, but clear evidence that vitamin D reduced both risk and mortality. Preclinical and transcriptomics studies provide compelling evidence that vitamins A and D combinations may be more effective for the prevention and treatment of breast and colon cancers, due to their significant synergistic effects and the larger number of cancer-signaling pathways impacted. **Conclusions**: Vitamins A and D reduce breast and colorectal cancer incidence, risk and mortality through multiple mechanisms of action, and offer significant potential as therapeutic and chemopreventative agents.

## 1. Introduction

Over the past three decades, data from in vitro, in vivo, and clinical trials have shown that vitamins play a crucial role in human health and disease prevention [1,2]. Both vitamins A and D have been reported to reduce the risk of cancer and improve prognosis, as well as enhance the quality of life during and post-chemotherapy [2].

**Figure 1:**
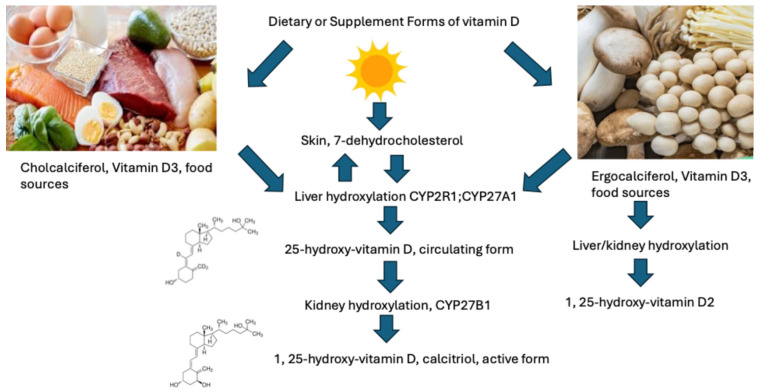
Sources of vitamin D and its metabolism to its active forms in the body.

Vitamins A and D are both fat-soluble nutrients and dietary compounds that are converted in the body to their respective active metabolites [2,3,4,5]. Vitamin D is technically a pro-hormone, that is biosynthesized in the skin from 7-dehydrocholesterol after direct exposure to sunlight (Figure 1, Ultraviolet B radiation, wavelength 290–315 nm) [1,4,5]. The chemical structure of vitamin D is very similar to other hormones, such as estradiol, cortisol and aldosterone, having the same cyclopentanoperhydrophenanthrene ring (Figure 1) [4,5,6]. Vitamin D occurs in two forms in the diet and supplements: vitamin D3 (VD3, cholecalciferol) that is synthesized from 7-dehydrocholesterol, and in the diet are obtained from fish and meat, as well as fortified milk. The second form, ergocalciferol (VD2) is produced by UV-B irradiation of the plant sterol, ergosterol, in nuts, seeds, legumes and fungi (mushrooms) (Figure 1). Structurally, VD2 differs from VD3 by one double bond between the C22 and C23 positions, as well as one methyl group at C24 in the side chain [3]. Both compounds are hydroxylated in the liver to form calcifediol (25-OH-D), the circulating but inactive metabolite. 25-OH-D is hydroxylated again in the kidneys to form the active metabolite 1,25-hydroxyvitamin D (1,25-OH-D), known as calcitriol (Figure 1). Both 1,25(OH)2D2 and 1,25(OH)2D3 have similar affinities for the vitamin D receptor (VDR), and the DNA binding sites of the vitamin D response element (VDRE) [3]. The VDR is expressed in both normal and malignant cells. Calcitriol (1,25-OH-D) binds to the VDR and then forms a heterodimer with the retinoid X receptor (RXR) with its bound ligand, 9 cis-retinoic acid [5]. This dimer binds to the vitamin D response elements or VDREs, along with several transcription factors, and initiates the transcription of vitamin D responsive genes. Thus, vitamin D initiated gene transcription, works in concert with vitamin A and its receptors to initiate gene transcription.

It is well understood that vitamin D is essential for the normal functioning of numerous cellular processes in the body, including cell growth and differentiation, glucose management, embryogenesis, skin and mucous membrane integrity, vision, immunity, and bone health [6,7,8,9]. Furthermore, vitamin D deficiencies have been associated with various forms of cancer [4,9].

**Figure 2:**
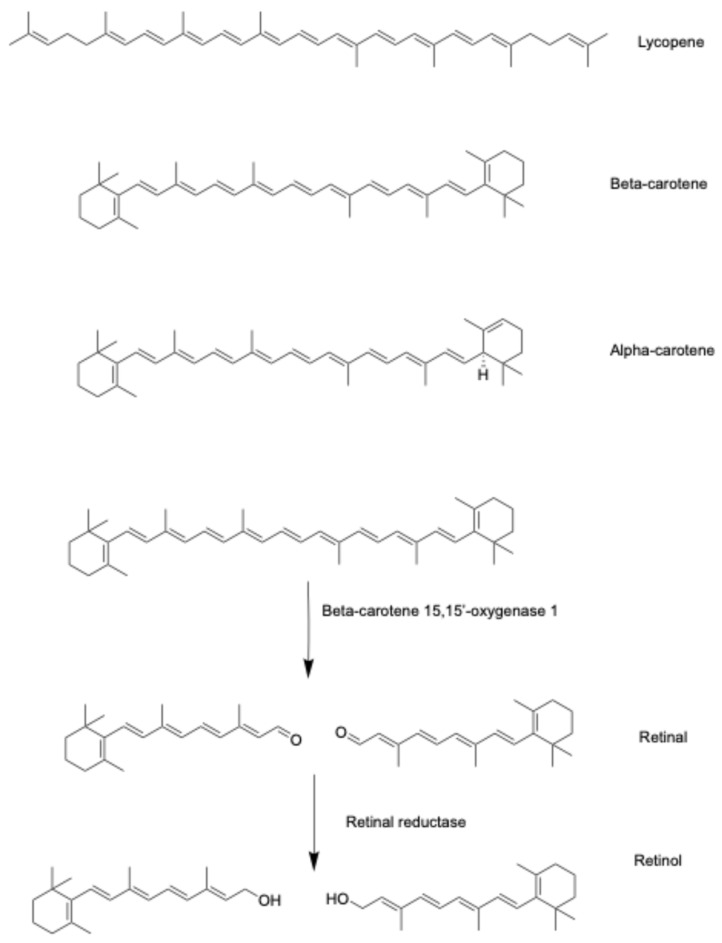
Carotenoids are the yellow, orange, and red fat-soluble pigments found in fruits and vegetables. Foods highest in carotenoids include carrots, sweet potatoes, pumpkin, cantaloupe, papaya, tomatoes, tangerines, winter squash, and dark leafy greens. It is estimated that there are approximately 600 carotenoid compounds, of which many carotenoids including lycopene, α-carotene and β-carotene are converted in the body to retinol and then further metabolized into the retinoids.

Vitamin A and its carotenoid precursors are fat-soluble compounds that include retinol, retinal, retinyl acetate, retinoic acids, as well as the α- and β-carotenes, among others (Figure 2) [10,11,12]. Dietary sources of vitamin A and the carotenoids include dairy products such as milk, yogurt, cheese, fatty fish, as well as yellow, orange and red fruits and vegetables. Vitamin A and the carotenoids are critically important micronutrients throughout a person’s lifetime, having numerous biological effects, including antioxidant, immune-enhancing, and anticancer activities [10,11,12]. Vitamin A is a required nutrient and must be ingested daily, as it is not synthesized in the body. Approximately 90% of dietary vitamin A is absorbed after ingestion; however, the carotenoids are poorly absorbed, with <3% being absorbed after ingestion [10,11,12]. In the body, both vitamin A and the carotenoids are converted to their active metabolites, retinal and retinoic acid. Retinoic acid has two conformations, trans and cis, that depend on the double-bond conformation; however, all-trans retinoic acid (ATRA) is the primary active metabolite and has the highest affinity for the retinoic acid receptor [10]. Unlike vitamin D, vitamin A has two nuclear receptors, the retinoic acid receptor (RAR) and retinoid X receptor (RXR) [13]. Retinoic acid serves as a ligand for both the RAR and the RXR, and upon binding, activates these receptors to regulate gene transcription.

The anticancer effects of vitamin A were first reported almost 100 years ago [14]. Since then, many researchers have demonstrated that all-trans retinoic acid (ATRA) was effective against leukemia and induced the differentiation of HL-60 leukemia cells, as well as inhibited the growth of gastric and breast cancers [14,15,16]. As an anticancer agent, ATRA is the most active isomer compared with the *cis*-retinoic acid isomer and exhibits stronger cytotoxic effects both in vitro and in vivo [15,16]. Vitamin D was also reported to have anticancer activities almost 85 years ago when Apperly [17] reported that populations living in Northern states in the USA had a higher cancer mortality than those living in Southern states. He further suggested that sunlight and vitamin D may also offer protection against breast, colon, and prostate cancers [17]. Later, in the 1980s, the effects of vitamin D and sunlight (UV radiation) on colorectal cancer (CRC) risk were described by Garland and Garland [18,19,20,21]. In 1989, these authors published a prevention study involving >25,000 volunteers, showing that CRC risk was reduced by 75% in volunteers with a serum 25-OH-D level in the range of 27–32 ng/mL and by 80% in patients with a higher serum level of 33–41 ng/mL [19]. Since then, population studies of the South and Southwest suggest that patients with a higher exposure to solar UVB radiation and vitamin D levels have reduced cancer incidence and mortality, as compared with populations living in the North [18,19,20,21]. The results from clinical, ecological, geographical, observational, and mechanistic studies further support these data, showing a higher risk of breast, colorectal, gastric adenocarcinomas, prostate, and other cancers is higher in patients with a vitamin D deficiency [18,19,20,21,22,23,24,25,26,27]. Thus, these data suggest that both vitamin A and D have a significant impact on cancer incidence, risk, prognosis, and mortality, most notably in cancers derived from epithelial tissues. Studies suggest that serum levels of these vitamins should be in at least a sufficient range for their anticancer activities.

The overall objective for this work was to review the clinical, epidemiological, and dose data from meta-analyses, as well as preclinical and next-generation sequencing (transcriptomics) data available for vitamins A and D and their combinations in breast and colon cancers.

## 2. Results and Discussion

### 2.1. Vitamin A Effects in Breast Cancer: Incidence, Risk, Metastasis, and Survival

Despite the suggested protective role of vitamin A and derivatives in breast cancer risk and progression, their clinical efficacy remains conflicting. Numerous studies show statistically significant reductions in breast cancer risk are associated with higher serum concentrations of retinol and α- and β-carotene [28,29,30,31,32,33,34,35]. In fact, these studies also reported that higher serum levels of α- and β-carotene may reduce the risk of metastatic breast disease by as much as 70% [28,29,30,31,32,33,34,35]. In addition, multiple epidemiological, cohort, and case–control studies reported that low serum concentrations or intake of vitamin A and/or carotenoids were associated with a higher breast cancer risk and increased metastatic disease [31,35,36,37,38,39,40,41], as shown in Table 1. In a pooled analysis, Eliassen et al. reported a significant reduction in breast cancer risk (~13–22% reduction) in women with higher levels of α-carotene, β-carotene, lutein, zeaxanthin, lycopene, and total carotenoids [40]. For women with higher β-carotene levels, there was a 48% reduced risk for ER-negative tumors [40]. In a follow-up nested case–control study, Eliasson et al. [41] measured plasma carotenoids using HPLC to determine the association between carotenoid concentrations and breast cancer risk. The results also showed that there was a significantly lower risk of breast cancer in women who had higher plasma concentrations of carotenoids, supporting their previous study [41]. More importantly, higher plasma concentrations of carotenoids were inversely related to breast cancer recurrence and death [41]. However, while these data are positive, results from older published studies contradicted these data and showed no effect [42,43,44,45], as shown in Table 1. These inconsistencies may be due to several factors, including the study methodology, variation in the types of vitamin A derivatives and doses used, methods of analysis, as well as pharmacological variations in absorption, metabolism, and bioavailability of the carotenoids [36]. Furthermore, randomized controlled clinical trials are usually limited to testing only one dose, as compared with measuring blood levels of vitamin A over a broad range of doses in observational studies.

Numerous meta-analyses have evaluated the case–control and cohort studies, as well as randomized controlled clinical trials [26,46,47,48,49,50], Table 1. A 2022 meta-analysis involving 49 case–control and cohort studies concluded that higher consumption of dietary vitamin A or supplements by women in North America and Asia reduced the incidence of breast, ovarian, and cervical cancers by 17% (*p* = 0.001), with breast and cervical cancers being the most significant [46]. Subgroup analysis conducted by the study type showed that while the cohort studies reported only a small risk reduction for all three cancers, the case–control studies showed a 25% risk reduction with higher consumption of vitamin A [46]. Further subgroup analysis of vitamin A administration through dietary measures and supplements, versus the use of vitamin A supplements alone, indicated that only high dietary vitamin A consumption, or diet plus supplements, significantly reduced cancer incidence, and not supplements alone [46]. Interestingly, a novel geographical subgroup analysis showed that higher dietary or supplement ingestion of vitamin A reduced the risk of these cancers in women in Asia (~50% reduction) and North America (~17% reduction), but this effect was not observed in women from either Europe or Oceania. Overall, this meta-analysis concluded that in Asia and North America, higher intakes of dietary vitamin A, with or without supplements, may significantly lower the incidence of breast and ovarian cancers [46].

One extensive review, covering 20 years of data (including 150 prospective cohort studies and nested case–control studies), investigated breast cancer risk and its association with serum concentrations of retinoids and carotenoids (Table 1) [50]. The results showed that higher blood levels or doses of retinol (and derivatives) were associated with a reduced risk of breast cancer in both pre- and postmenopausal women [50], as shown in Table 1. Furthermore, the study concluded that higher concentrations of α- and β-carotene in the serum or plasma significantly reduced breast cancer metastasis and improved survival rates. However, the analysis also noted that several studies reported little or no effect of vitamin A derivatives on breast cancer risk, thereby indicating that there are inconsistencies in the clinical data [50]. These inconsistencies may be due to several limitations, including the study design and methodology, variation in the types of vitamin A derivatives used and doses, different methods of analysis, as well as pharmacological variations in absorption, metabolism, and bioavailability. It has also been suggested that the methods for measuring serum vitamin A levels in some studies may have been a problem, and using stable isotope dilution methods may give a better indication of the total-body and liver vitamin A reserves and is recommended in future studies on vitamin A status [51,52,53].

Several meta-analyses investigated the association between vitamin A and carotenoid ingestion with plasma concentration and overall breast cancer survival (Table 1) [47,48,49]. A systematic review and meta-analysis investigated the association between breast cancer and overall survival with dietary ingestion of vitamin A or supplementation [48]. This analysis concluded that higher dietary intake of β-carotene increased breast cancer survival by 30%, but other vitamin A derivatives, including α-carotene, β-cryptoxanthin, lycopene, retinol, and lutein, had no effect on overall survival [48]. In a 2021 meta-analysis, Li et al. [49] analyzed eight studies that used antioxidants, including vitamins A, E, or C post-diagnosis, to determine the association with breast cancer (BC) survival. No significant association was found for vitamin A or E use, and only vitamin C intake after BC diagnosis was significantly associated with better overall survival (Table 1) [49].

Overall, data generated from more multiple studies suggest a correlation between vitamin A and carotenoid intake with the risk and survival rates of breast cancers. Several studies indicated that higher dietary consumption of vitamin A, particularly in Asia and North America, reduced the incidence of BC, although the effects of supplements alone remain unclear. Limitations and inconsistencies were observed across studies, due to variations in study design and methodology, confounding with other dietary components, sampling issues, the types of vitamin A derivatives used, methods used for retinol measurement, and the heterogenicity of breast cancer itself. Thus, the supporting evidence for vitamin A has many limitations, and further research, including randomized controlled clinical trials (RCTs) with improved methodologies, is needed to prove causality in breast cancer.

### 2.2. Vitamin D Effects in Breast Cancer: Risk, Prognosis, Metastasis, and Survival

Breast and early-stage colon cancer tissues both contain very high levels of the vitamin D receptors, suggesting a role for vitamin D in BC and CRC development. Lower serum levels of vitamin D have also been associated with infections, poor skeletal health, and an increased breast cancer risk, resulting in a poorer prognosis [54,55,56,57], Table 2. A 2018 meta-analysis of 68 observational studies (30 case–control, 21 nested-case–control and 17 cohort studies) assessed exposure to calciferol (vitamin D), calcifediol (25-OH-D), and calcitriol (1,25-OH-D) with BC risk [56]. Analysis of the case–control and cohort studies found a reduced risk of BC associated with higher serum levels of 25-OH-D. Results from the case–control studies showed a reduced risk for BC in premenopausal women only. No significant risk reduction was associated with the ingestion of vitamin D (calciferol) or 1,25-calcitriol or supplements [56].

One large 2019 systematic review and meta-analysis of 22 observational studies, including 229,597 participants, evaluated the evidence for vitamin D levels in diet and 25-OH-D levels in serum, with BC occurrence in both premenopausal and postmenopausal women [57]. The results of this study showed that there was a direct association between BC occurrence and a 25-OH-D deficiency (Table 2). The results from this study were limited by the use of only three public databases, as well as the inclusion of observational and low-powered studies [57].

A prospective cohort study involving 3995 women measured serum 25-OH-D levels at the time of BC diagnosis and then followed up for ~10 years [58], Table 2. The serum concentrations of vitamin D were designated as <20 ng/mL deficient, 20–30 ng/mL as insufficient, and ≥30 ng/mL as sufficient. Correlation of the vitamin D concentrations with overall survival, BC-specific survival, recurrence-free survival, or invasive disease-free survival was performed. Overall, African American women with BC had the lowest vitamin D levels compared with other ethnic groups. Post-follow-up data showed that women who had a sufficient 25-OH-D serum concentration at diagnosis had a significantly better survival outcome, as well as a better BC prognosis. The study concluded that maintaining sufficient 25-OH-D levels was important for women with breast cancer, especially for women of African descent and patients with advanced disease [58]. Some of the limitations of this study included an ethnically diverse population, heterogenicity of BC and diagnoses, potential confounding by other co-morbidities, and measurement methods.

Interestingly, a 2022 retrospective study involving data from 292 women newly diagnosed with BC reported that ~63.7% of patients had 25-OH-D levels of <30 ng/mL (deficient/insufficient), and 56% of these women were <65 yrs of age (Table 2) [59]. Many of these young women were obese, and patients with deficient/insufficient 25-OH-D levels presented with more ductal tumors (*p* < 0.014), increased grade III tumors (*p* < 0.01), as well as ER- and triple-negative breast cancers (*p* = 0.04). Triple negative BC was five-fold higher in patients with deficient/insufficient levels of 25-OH-D. The study concluded that deficient/insufficient 25-OH-D levels were associated with more aggressive breast cancers, as well as ER-negative cancers [59]. The limitations of this study included the heterogenicity of BC and different BC staging, varied methods for the measurement of 25-OH-D levels, and vitamin D levels were only measured at diagnosis.

Mackey et al. [60] investigated the effects of vitamin D deficiency on BC risk, as well as the impact of menopause on vitamin D-associated breast cancer risk (Table 2). The study used the TriNetX platform to generate cohorts of patients that were categorized based on the International Classification of Disease-10 codes. The cohorts included women > 40 yrs of age with no breast cancer history, with a diagnosis of menopause, and one cohort with ≤20 ng/mL of 25-OH-D (deficient), and the second cohort had >30 ng/mL (sufficient). There were 73,659 women in each arm, and the arms were balanced for age, race, and ethnicity, and evaluated breast cancer risk. Two other pre-menopausal cohorts were also created using the same parameters for 25-OH-D levels and cohort balancing. The results of this study showed that a vitamin D deficiency in postmenopausal women was significantly associated with a 45% increased risk of BC (*p* < 0.0001), as compared with women who had sufficient levels. The increased risk was also significant for pre-menopausal women who were vitamin D deficient, with an ~18% higher risk (*p* < 0.0001). The study concluded that deficient vitamin D levels in both premenopausal patients and postmenopausal patients were associated with a significantly higher risk of BC [60]. This study used a diverse population of pre- and post-menopausal women with heterogeneous BC diagnoses and stratified them using the TriNetX platform, a novel method that had not been used before.

More recently, a 2024 meta-analysis reviewed six studies that investigated the correlations between 25-OH-D levels and breast cancer progression and chemotherapeutic resistance [61]. The results of this meta-analysis concluded that sufficient 25-OH-D levels were associated with a 22% reduction in non-response to neo-adjuvant chemotherapy (NACT), and a 35% reduction in the risk of BC progression. The study further recommended that BC patients with low 25-OH-D levels should be rapidly treated to increase these levels into the sufficient range during BC treatment [61]. Furthermore, a recent pooled analysis of six cohort studies, involving 1291 patients with breast cancer, showed a significant correlation between pretreatment plasma 25-OH-D levels and the patient response to NACT (Table 2) [62]. The study concluded that there was a significant beneficial effect between the pretreatment of vitamin D deficiencies in women with BC and their response to NACT [62]. The study also suggested that women with BC should be immediately treated for their 25-OH-D deficiency/insufficiency at diagnosis, as well as during NACT treatment. The results of this study are limited by the inclusion of patients with cancer in stages I, II, and III, as it is well known that vitamin D receptor levels in breast tissues decline in later stage BC.

In summary, meta-analyses of observational, case–control, nested-case–control, cohort, and randomized controlled clinical studies published between 2018 and 2025 investigated the relationship between 25-OH-D levels and breast cancer risk, prognosis, treatment outcomes, and survival. These meta-analyses have consistently concluded that a vitamin D deficiency was associated with an increased risk of developing breast cancer in both premenopausal and postmenopausal women. Furthermore, sufficient circulating 25-OH-D levels have been linked to improved survival outcomes, lower tumor grades, and less ER- or triple negative breast cancers in newly diagnosed patients. These data highlight the importance of maintaining sufficient vitamin D levels, especially in women of African descent and those with advanced disease or obesity. While some studies addressed vitamin D intake through diet and supplements, the serum concentrations of 25-OH-D appeared to be a significant factor in BC development and outcomes. Daily vitamin D ingestion may also reduce cancer mortality, highlighting the importance of maintaining adequate vitamin D levels for prevention and treatment. The limitations of these studies include considerable variability in the types and quality of literature used, differences in methodologies and standardization across studies, methods of statistical analyses, as well as different cutoffs for vitamin D levels. Some studies were limited to data from only three public databases, with no cross-referencing, and used low-powered studies, as well as evidence from observational studies.

### 2.3. Effects of Vitamin D in Colorectal Cancer (CRC): Risk, Prognosis, and Survival

In general, data for the association between vitamin A and colorectal cancer (CRC) risk have been inconsistent or negative [63,64]. However, numerous studies from multiple investigators have shown that the risk, incidence, recurrence, and mortality of colorectal adenomas were inversely related to vitamin D intake, and that increased serum 25-OH-D levels were better for the overall prognosis and survival rate (Table 3) [65,66,67,68,69,70,71,72,73,74,75,76,77,78,79,80,81]. A 2023 meta-analysis evaluated 28 case–control and prospective cohort studies to determine the correlation between the risk of CRCs with the serum or plasma levels of 25-OH-D [72], as shown in Table 3. The results of this meta-analysis showed that higher levels of 25-OH-D were associated with a 39% reduction in CRC risk in the case–control studies, while a 20% risk reduction was observed in the cohort studies. Interestingly, these results were only statistically significant for women and not for men [72]. Similar results (~25% risk reduction for CRC) were reported in two earlier meta-analyses [73,74].

In terms of CRC incidence and progression, a 2020 systematic review and meta-analysis of 166 studies involving >800,000 cases reported that both vitamin D and calcium intake, as well as serum 25-OH-D levels, were inversely associated with a reduced incidence of CRC, especially in women [75], as shown in Table 3. Higher serum levels of 25-OH-D also increased overall and CRC-specific survival, and European and US populations benefited the most [75]. One 2024 systematic review and meta-analysis investigated the association between 25-OH-D levels prior to chemotherapy to the time to the development of metastatic disease (Stage 4) [76], as shown in Table 3. The results showed that patients with lower circulating levels of 25-OH-D prior to the initiation of chemotherapy had a 47% increased risk of death, and a 38% increased risk of developing metastatic CRC [76]. However, the study did not include the chemotherapy regimens or prognostic factors such as the initial disease burden, treatment responses, and toxicity. A 2019 meta-analysis of 15 randomized controlled trials (RCTs) that correlated vitamin D supplementation with cancer incidence and mortality [77]. Ten of the trials investigated the effects of vitamin D on cancer incidence, and five trials that investigated cancer mortality were included. The results showed that while cancer incidence was not impacted, even when serum 25-OH-D levels exceeded 100 nmol/L (40 ng/mL), there was a significant 13% decrease in cancer mortality and 7% reduction in total mortality over 3–10 years of follow-up, that was attributed to daily and not bolus dosing of vitamin D [77], Table 3.

One large meta-meta-analysis of 35 meta-analyses, including observational studies and randomized controlled clinical trials, investigated the relationship between cancer outcomes, cancer incidence and mortality, with 25-OH-D levels and vitamin D intake [78], Table 3. Analysis of the observational studies showed that higher serum 25-OH-D concentrations and vitamin D intake significantly (*p* < 0.001) reduced cancer risk and cancer-related mortality (*p* < 0.001). However, the analyses of the randomized controlled trials showed no significant relationship between the risk of cancer and the administration of single specific doses of vitamin D [78]. Limitations of these studies included the fact that cancer incidence and cancer-related deaths were not the primary endpoints of many of these RCTs. Also, the RCTs did not measure 25-OH-D levels at the conclusion of the studies. Two other meta-analyses of RCTs published in 2022 reported conflicting results, with one (12 RCTs) reporting that vitamin D supplementation did not reduce overall cancer mortality [79]; while the other (26 RCTs) reported a significant reduction in overall cancer mortality by 10%, especially in combination with calcium supplementation [80], Table 3. The differences in the results of these studies may be due to the number of RCTs included in the final analyses and the statistical methods used for the analyses (Table 3). Finally, a 2023 systematic review and meta-analysis assessed the effects of vitamin D on cancer mortality using data from 14 RCTs, as well as individual patient data from 7 of the RCTs [81]. This study suggested that vitamin D3 reduced total cancer mortality, but the results were not statistically significant. However, a subgroup analysis showed that daily intake of vitamin D3 reduced cancer mortality by 12% as compared with placebo, while a bolus dose of vitamin D showed no effect [81].

In summary, while the evidence associating vitamin A intake and levels on CRC remains inconclusive, the reviewed data strongly support a correlation between higher 25-OH-D levels and a reduced risk of colorectal cancer, particularly in women. Although observational studies consistently show these benefits, data from RCTs offer a more complex picture regarding the impact of supplementation on overall cancer outcomes. Evidence from RCTs suggests that daily vitamin D3 supplementation may reduce cancer mortality, and this effect may be synergistic with calcium. Furthermore, higher vitamin D levels appear to be associated with better overall and CRC-specific survival rates, as well as a lower risk of metastatic disease and recurrence. Daily dosing also appears to be a critical factor in the effectiveness of vitamin D supplementation for survival benefits. It should be noted that the RCTs analyzed had many limitations, including different analytical methods and cutoffs for vitamin D levels. Often, vitamin D concentrations were expressed using different measurement units. The chemotherapy regimens were also not discussed in some RCTs, nor were prognostic factors such as the initial disease burden, treatment responses, or toxicity. The main limitation was that the primary outcomes for many of the RCTs were not designed to test the hypothesis that vitamin D influenced the risk of cancer incidence or mortality, as these were secondary outcomes.

### 2.4. Vitamin A and D Dosing in Cancer

#### 2.4.1. Vitamin D Dosing in Cancer

Although still controversial, it is commonly accepted that patients have a vitamin D deficiency if they have a serum 25-OH-D concentration < 50 nmol/L (20 ng/mL), and serum levels of 25-OH-D are still considered insufficient at a concentration of 51–74 nmol/L (21–29 ng/mL) [82,83]. Serum concentrations ≥ 30 ng/mL are considered sufficient for general health (Table 4) [82,83]. However, the optimal serum concentrations of 25-OH-D needed for antineoplastic effects have not yet been defined. Several studies have suggested that for the anticancer effects, serum 25-OH-D concentrations should be at least in the sufficient range of 75 nmol/L (30 ng/mL), or higher, 90 to 110 nmol/L (~36–50 ng/mL) (Table 5) [84,85,86,87,88]. In 2006, Giovannucci et al. [84] developed a linear regression model to estimate the serum 25-OH-D levels using data from 1095 men to predict the 2-OH-D levels in 47,800 participants in the Health Professionals Follow-up study. Prospective examination of this model in relation to cancer risk showed that an increase of 25 nM/L in the predicted level was associated with a 17% reduction in cancer incidence, a 29% reduction in cancer mortality, and a 45% reduction in gastrointestinal cancers in men [84]. Thus, current recommended daily allowances (Table 4) may be insufficient to prevent serious diseases such as cancer.

In a 2009 study, Crew et al. [85] investigated the prevalence of a vitamin D deficiency in 103 premenopausal women with BC who started adjuvant chemotherapy and participated in a year-long zoledronate intervention trial (Table 5). The results of this study show that at baseline, 86% of pre-menopausal women diagnosed with BC (and during chemotherapy) had deficient or insufficient 25-OH-D levels. This deficiency was observed in 66% of Caucasian women, 84% of Hispanic women, and 80% of women of African descent [85]. Administration of calcium/vitamin D supplements, in a dose of 1000 mg/400 IU/day, for one year, was not adequate to increase the serum vitamin D levels to ≥30 ng/mL (sufficient). Only 15% of Caucasian or Hispanic women achieved sufficient serum 25-OH-D concentrations with 400 IU/day of vitamin D, and no women of African descent achieved sufficient levels [85]. These data indicate that higher doses of vitamin D are needed to increase the 25-OH-D levels in premenopausal women with BC to attain a sufficient 25-OH-D serum level.

Khan et al. [86] conducted a clinical trial involving 40 post-menopausal women with early-stage hormone-positive BC to determine the effects of high-dose vitamin D supplementation on the musculoskeletal adverse events associated with letrozole therapy (Table 5). The median 25-OH-D serum concentration in these patients was 23 ng/mL at the beginning of the trial. During the study, each woman was administered 50,000 IU/week of vitamin D3 for 12 weeks, assessed at 6 and 12 weeks, and then given a daily maintenance dose of 600–1000 IU for another 3–6 months. High-dose vitamin D (50,000 IU/week) increased serum vitamin D levels to >40 ng/mL over a six-week period in 98% of the participants on letrozole therapy. However, the follow-up showed that the maintenance dose of vitamin D3 (600–1000 IU/day) was not high enough to maintain sufficient 25-OH-D levels, and these levels declined at a rate of ~7% per month. The study concluded that supplementation with the recommended doses of 600–1000 IU of vitamin D3/day was not as effective as high-dose administration of vitamin D3 (50,000 IU/week) for maintaining 25-OH-D levels > 40 ng/mL in post-menopausal women with early-stage breast cancer (Table 5) [86]. No adverse effects were reported.

One retrospective study measured the serum 25-OH-D concentrations in 224 women with breast cancer (stage 0-III) at diagnosis [87]. The women were retested for 25-OH-D levels after treatment with vitamin D at a daily low dose of 1000 IU/day or a weekly high dose of 50,000 IU/week for 8 to 16 weeks (Table 5). Measurement of 25-OH-D levels at the beginning of the study showed that a high percentage (66.5%) of women had either insufficient (20–31 ng/mL) or deficient (<20 ng/mL) levels of 25-OH-D. A significant (*p* < 0.05) percentage of women with later-stage disease, who had radiation therapy or who were not Caucasian, had a vitamin D level that was either deficient or insufficient [87]. Women with insufficient levels were treated orally with low-dose vitamin D (1000 IU/day), while women who were deficient received high-dose vitamin D (50,000 IU/week), and women with sufficient vitamin D levels were not treated. After 8–16 weeks of treatment, the levels of 25-OH-D significantly (*p* < 0.01) increased in women in the high dose group, but no significant change in 25-OH-D levels was observed in women in the low dose group. This study suggested that a dose of 1000 IU of vitamin D per day was not high enough to increase the serum 25-OH-D levels in women with insufficient levels of vitamin D [87]. One of the issues with this study was the cancer staging. It is well known that there is a loss of the vitamin D receptor in the higher stages of BC, and this may impact clinical outcomes.

McDonnell et al. [88] have suggested that the risk of breast cancer was significantly reduced by higher serum levels of 25-OH-D. Analysis of data extracted from two RCTs and a prospective cohort study involving women over the age of 55 concluded that patients with serum concentrations of 25-OH-D (>60 ng/mL) had an 82% lowered incidence of breast cancer and 78% lowered risk for breast cancer as compared with women who had 25-OH-D levels of <20 ng/mL, even after adjusting for other multiple variables [88]. Similarly, results from another study suggested that 25-OH-D concentrations of >40 ng/mL may also be protective [89].

#### 2.4.2. Vitamin A Dosing in Cancer

RCTs investigating vitamin A supplements for cancer chemoprevention and treatment have reported that large doses of vitamin A are well tolerated and have a significant effect on skin cancer outcomes (Table 6) [90,91,92,93]. In a randomized, placebo-controlled dose-escalation trial, retinyl palmitate was administered at a dose of 25,000, 50,000, or 75,000 IU/day to 129 volunteers with severe sun-damage to the skin on their arms for one year [90]. The results of this study reported no toxicity as compared with the placebo. Skin biopsies from 113 patients showed a significant karyomeric improvement and dose response for 25,000 and 50,000 IU/day. Increases in the retinoic acid receptor were also observed at the 50,000 IU/day dose [90]. A randomized phase clinical III trial, involving 2297 patients with actinic keratosis, investigated the effects of retinol at a dose of 25,000 IU/day for 5 years. While there was a slight elevation of serum cholesterol, no other toxic effects, and a 32% reduction in the risk of squamous cell carcinoma was observed [91]. Furthermore, results from a phase I study reported that vitamin A doses of >350,000 IU/day were generally tolerated in patients with various advanced cancers, with some adverse events reported (Table 6) [93]. One study involving ~4700 patients admitted to a long-term hospital care facility reported that most patients admitted were vitamin D deficient/insufficient [94]. Subgroup analysis of 777 patients revealed that 92.8% had either deficient or insufficient levels of vitamin D [94]. Due to the length of their stay in the hospital and lack of exposure to sunlight during that time, they were administered oral daily doses of vitamin D in the range of 5000 IU to 50,000 IU/day based on their disease status. No cases of hypercalcemia were reported. The study concluded that administration of 5000 IU to 50,000 IU/day of vitamin D per day to long-term hospitalized patients was safe [94].

Overall, multiple studies by different researchers have tested varying doses of vitamin D in cancer patients to determine an optimal vitamin D supplementation strategy. The results showed that serum 25-OH-D levels ≤ 20 ng/mL are consistently associated with increased risk of both breast and colon cancers. Sufficient levels of 25-OH-D (≥30 ng/mL) are needed to reduce risk and mortality, but higher serum levels (40–60 ng/mL) may also further reduce risk. These studies also indicate that the current recommended daily doses of 400–800 IU/day (Table 7) are inadequate, and higher doses are needed to increase or maintain adequate vitamin D levels in cancer populations. Higher oral doses, such as 50,000 IU/week, may be needed to achieve and sustain sufficient vitamin D levels, particularly for women undergoing cancer treatment, women of color, and those with specific risk factors, such as obesity and diabetes. High doses of vitamin A (25,000–75,000 IU) appear to be well tolerated, while very high doses are associated with adverse events (Table 6). The outcomes of these studies suggest that higher doses of vitamin A and D are well tolerated and may be needed to address deficiencies in patients with cancer.

### 2.5. Supporting Preclinical and Molecular Studies

#### 2.5.1. Vitamins A and D Impact Molecular Signaling and Induction of Apoptosis

**Figure 3:**
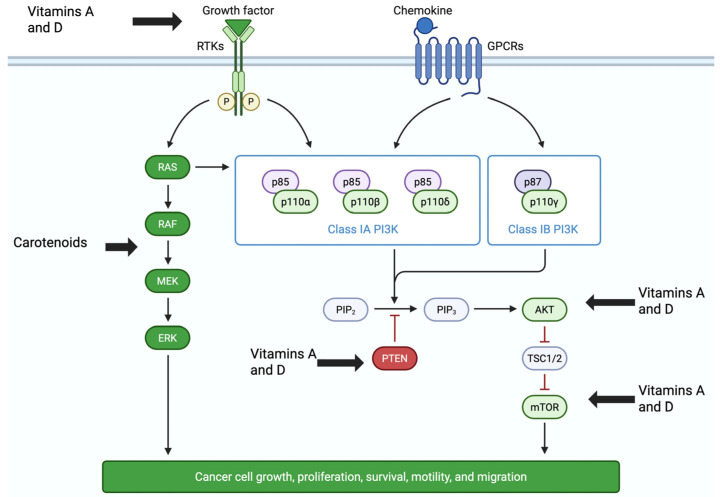
Effects of vitamins A and D on the expression of genes in the PI3K/Akt/mTOR and RAS/RAF/MEK/ERK1/2 signaling pathways. Created in BioRender. Mahady, G.B. (2025). www.Biorender.com/53jghnk green colors represent genes or pathways downregulated, while the red colors represent genes upregulated.

Many molecular signaling pathways are dysregulated in cancer cells, allowing them to grow, survive, alter their phenotype, and invade other tissues. Activation of receptor tyrosine kinases by growth factors increases signaling through the phosphoinositide 3-kinase (PI3K)/protein kinase B (AKT)/mammalian target of rapamycin (mTOR) (PI3K/Akt/mTOR; PAM) pathway (Figure 3), increasing cancer cell proliferation and metastasis [95]. The activation of PAM signaling is often observed in human cancers and may increase chemotherapeutic resistance, as well as promote phenotypical changes, such as the epithelial–mesenchymal transition, thereby increasing metastatic disease [95]. Increased signaling through the PAM pathway alters the expression and activation of the Bcl-2 family of proteins in cancer cells, which regulate programmed cell death (apoptosis) [96]. PAM signaling upregulates the expression and activation of the anti-apoptotic proteins, Bcl-2, and Bcl-xL, while the pro-apoptotic proteins, including Bax, Bad, and the tumor suppressor p53, are downregulated [96]. Another dysregulated pathway in cancer cells includes the RAS/RAF/MEK/ERK1/2 pathway [97]. This pathway regulates gene expression of c-Myc, c-Jun, c-Fos, and nuclear factor-kappa-beta (NF-κβ) [96]. Activation of NF-κβ leads to the transcription of inflammatory cytokines and other genes, such as interleukin-6 and -8, cyclooxygenase-2, and iNOS [97]. Increased phosphorylation of the extracellular signaling-regulated kinase1/2 (ERK1/2) can further activate NF-κβ signaling, thereby increasing the expression of anti-apoptotic proteins, including Bcl-2, and thereby reducing cancer cellular apoptosis [96,97].

Many in vitro and in vivo mechanistic studies have demonstrated the wide range of anticancer activities of vitamin A or D derivatives and metabolites [65,98,99,100,101,102,103,104,105,106,107,108,109,110,111,112,113,114]. These include inhibition of cell proliferation, induction of differentiation or apoptosis, inhibition of angiogenesis, as well as suppression of invasion and metastasis in a wide range of cancers. Studies have shown that vitamins A or D (individually) have significant antiproliferative and apoptotic effects in cultured breast, colon, gastric, leukemia, and pancreatic cancer cells [98,99,100,101,102,103,104,105,106,107,108,109,110,111,112,113,114]. For example, calcitriol (1,25-OH-D) induced apoptosis in colorectal adenomas by up-regulating pro-apoptotic proteins, including Bax, down-regulating the anti-apoptotic protein Bcl-2, and inducing p53-independent apoptosis [108]. In acute myeloid leukemia cells, treatment with all-trans-retinoic acid reduced cell proliferation by increasing p53 and cell cycle arrest [98]. Furthermore, synergistic effects have been reported when vitamin A or D was combined with 5-fluorouracil or metformin, showing increased cytotoxicity and apoptosis in cancer stem cells, as well as breast and colon cancers [112,113,114]. Both vitamins A and D have been reported to downregulate Bcl-2 and Bcl-xL mRNA expression, as well as upregulate Bax, Bak, p53, and caspase mRNA expression and activities, leading to apoptosis in numerous types of cancer cells [105,106,107]. In breast cancer cells, carotenoid derivatives induced apoptosis by suppressing the PI3K/Akt/mTOR and RAS/RAF/MEK/ERK1/2 signaling pathways [50], Figure 3.

#### 2.5.2. Vitamins A and D Are Antioxidants and Reduce Reactive Oxygen Species (ROS)

Other mechanisms of cancer include the generation of reactive oxygen species (ROS), formed by oxidative phosphorylation in the mitochondria of cells. ROS have long been associated with the initiation and progression of cancers due to their ability to damage DNA, protein, and lipids [93,115,116,117,118]. In cancer cells, ROS production is increased, leading to enhanced DNA damage and increased oncogenic signaling [116,117]. Increased ROS accumulation has been reported to suppress apoptosis, increase cancer cell proliferation, metastases, and angiogenesis, as well as increase resistance to chemotherapy [116,117]. Carotenoids have been reported to reduce NF-κβ expression and reactive oxygen species (ROS) formation and activity, leading to reduced inflammation [93]. Thus, since vitamins A and D are both potent antioxidants that can reduce ROS accumulation and DNA damage, it is reasonable to expect that reducing ROS production in cells is one of the mechanisms by which they exert their anticancer activities.

#### 2.5.3. Vitamin D Induction of Autophagy

Beyond apoptosis, autophagy is another canonical pathway well known to be associated with tumor suppression [118,119,120,121]. Autophagy is a lysosome-regulated pathway necessary for cell differentiation, growth, homeostasis, and overall survival. This pathway is responsible for the catabolism and removal or recycling of unwanted or damaged cellular components [118,119,120]. During oxidative stress and apoptosis, autophagy may be initiated to regulate inflammation, immunity, and cell growth [118,119,120]. Both vitamin D and the vitamin D receptor (VDR) are associated with the induction of autophagy by regulating calcium absorption and release [120]. Vitamin D3 initiates autophagy during oxidative stress by increasing calcium release from the endoplasmic reticulum and downregulating the mammalian target of rapamycin (mTOR) expression [120]. In addition to mTOR, the Bcl-2 family of proteins is also involved in autophagy [118,119,120]. During endoplasmic reticulum stress, transcription and activation of two enzymes, inositol-requiring enzyme 1 and protein kinase RNA-like endoplasmic reticulum kinase (PERK), phosphorylate Bcl-2 and Bcl-XL (two autophagy inhibitory proteins) and deactivate them, thereby inducing autophagy [118,119]. Activation of PERK further increases the initiation of autophagy by increasing the transcription and activation of both the C/EBP homologous protein and activating transcription factor 4, resulting in mTOR inhibition and autophagy [118,119,120].

#### 2.5.4. Vitamins A and D Downregulate Estrogen Signaling Pathways

Other pathways that may be dysregulated include the estrogen signaling pathways. Estrogens are well known to play a central role in hormone-positive cancers; thus, treatments that reduce estrogen pathway signaling, particularly in breast cancer, are commonly used in prevention and treatment [121,122,123]. It is well known that estrogen receptor 1 (ERα) agonists, such as 17β-estradiol (E2), increase breast cancer cell proliferation. Treatment with the vitamin A derivative, all-trans retinoic acid, inhibited the proliferation of the ERα-positive cell lines MCF-7, MCF7/BUS, and the transgenic line U2OS-ERα-Luc [121]. In BC, ERα and retinoid receptor (RAR/RXR) signaling pathways had opposite effects, and activation of the RAR/RXR reduced BC cell growth, while activation of ERα signaling increased BC cell proliferation [121,122]. In addition to vitamin A, the active form of vitamin D, calcitriol, downregulated the expression of the estrogen receptor 1 and aromatase (CYP19A1) genes, an enzyme that synthesizes estrogen from androgenic precursors in MCF-7 cells [123]. Suggesting that both vitamin A and D have anti-estrogenic effects.

### 2.6. Transcriptomic and Proteomics Studies of Vitamins A and D in Colon and Breast Cancers

Cancer is a complex, heterogeneous group of diseases that continues to present significant treatment challenges. The vast array of biological alterations occurring within cancer cells is driven by numerous genetic mutations and molecular changes and underscores the need to decipher the mechanisms underlying cancer initiation, progression, and the treatment response [124,125]. Alterations in RNA and protein expression, along with molecular changes induced by cancer therapies, are essential for understanding both therapeutic efficacy and mechanisms of action.

The transcriptome is defined as the complete set of all transcribed RNA molecules, including protein-coding mRNAs and non-coding RNAs (such as microRNAs and long non-coding RNAs), that are expressed in the genome of cells at any given time [124,125]. Transcriptomic analysis measures the complete set of RNA transcripts (the transcriptome) within a cell to understand gene expression levels and their regulation. Common methods used for transcriptomic analyses include next-generation or deep sequencing (RNA-Seq), coupled with bioinformatic analyses, that provide a high-resolution snapshot of the entire transcriptome. Thus, transcriptomic analysis provides important insights and understanding into the biological processes, disease mechanisms, and responses to environmental stimuli or treatments by detecting dynamic changes in gene expression over time.

The effects of vitamin D on the transcriptome of numerous cancer cells, including colon, prostate, and breast cancers, have been investigated using multiple techniques, including RNA-seq and microarrays [124,125,126,127]. In various cancer cell lines, treatment with vitamin D regulated cell cycle and proliferation by downregulating the expression of cell cycle and oncogenes and upregulating the level of transcripts associated with apoptosis and tumor suppression expression (described in Table 8) [124]. In addition to vitamin D, vitamin A derivatives have also been reported to induce significant alterations in the transcriptome in cancer cells [125,126,127]. In two triple-negative breast cancer cell lines, Coyle et al. [125] showed that treatment with retinaldehyde dehydrogenase 1A3 (ALDH1A3) expression or all-trans retinoic acid (ATRA) induced differential gene expression profiles with minimal overlap. The expression of numerous ATRA-inducible genes associated with inflammation and immunity was reported (Table 8) [125]. Since retinoic acid treatments can also result in epigenetic modulation, these authors further found that DNA methylation impacted the inducibility of some ATRA-responsive genes, while histone acetylation played a limited role [125]. The pro- or anti-tumor effects of ATRA in these cell lines were not mediated by the peroxisome proliferator-activated receptor but involved retinoic acid response element-independent pathways. The transcription factor interferon regulatory factor 1 (IRF1) was upregulated by ATRA and epigenetically regulated and was necessary for full ATRA induction of the gene for cathepsin S in MDA-MB-231 cells. These results indicated that an ATRA impacted a complex network of signaling responses in breast cancer cells [125]. In a 2016 study, Carrier et al. [126] analyzed the phospho-proteomic and transcriptomic effects of retinoic acid (RA) in RA-responsive MCF-7 and RA-resistant BT-474 breast cancer cells [126]. Treatment with RA induced the phosphorylation activation of mammalian STE20-like protein kinase 4 in MCF7 cells, as well as the Ppp4r3a protein phosphatase 4 regulatory subunit 3A (SMEK1) in BT-474 breast cancer cells (Table 8). RA-treatment further increased the concentration of phosphoproteins in BT-474 cells and altered phosphorylation of proteins associated with mRNA processing and DNA repair [126]. In MCF-7 breast cancer cells, phosphorylation of the retinoid receptor RARα sites S74 and S77 was observed, but this was not seen in BT-474 cells. Transcriptomic analysis showed that the expression of ~40% more genes was altered in RA-treated MCF-7 cells than in BT-474 cells. The results of this study suggested that RA resistance in BT-474 cells may be caused by altered phosphorylation of proteins associated with mRNA processing, reduced RARα phosphorylation, and altered expression of RA target genes [126]. Interestingly, a 2021 clinical study reported that oral administration of vitamin D increased the plasma concentrations of 25-OH-D, and this resulted in alterations in differential gene expression (as determined by transcriptomic analysis) in the rectal mucosa that corresponded with anti-tumor activities [127]. Thus, recent advances in next-generation sequencing (transcriptomic analysis), proteomics, and other high-throughput multi-omics technologies have significantly improved our ability to characterize the molecular impact of cancer treatments. Using these cutting-edge analytical tools in clinical settings can significantly enhance our understanding of the molecular impact of vitamin A and D treatments for breast and colorectal cancers.

### 2.7. Synergistic Effects of the Combination of Vitamins A and D and Transcriptomic Analyses in Breast and Colon Cancers

In recent years, multiple-targeted integrative approaches to cancer have hypothesized that using combinations of treatments may be more effective for cancer prevention and treatment [128,129,130]. For example, synergistic effects have been demonstrated in 1,25-OH-D (calcitriol, Vitamin D3) treated UF-1 cells, a retinoic acid-resistant acute promyelocytic leukemia cell line [98]. The results showed that the addition of 1,25-OH-D induced differentiation of this cell line by altering cell cycle-induced blockade through p21 and p27 signaling [98]. In the liver cancer cell line HepG2, treatment with a combination of all-trans-retinoic acid (ATRA) and 1,25-OH-D induced apoptosis through the upregulation of the p21 and p27 mRNA and protein expression and was more effective than either vitamin alone [131]. In cultured PC3 prostate cancer cells, treatment with a combination of ATRA and 1,25-OH-D was synergistic, and increased apoptosis by enhancing BAX protein expression, as well as significantly downregulated cell cycle genes such as cyclin D1 protein expression and inhibited the G0 phase of the cell cycle [132]. In cultured breast, colon, and gastric cancer cells, combinations of ATRA and vitamin D2 and D3 synergistically reduced cell viability, as well as induced apoptosis and autophagy, by impacting numerous canonical signaling pathways [133,134,135,136,137].

**Figure 4:**
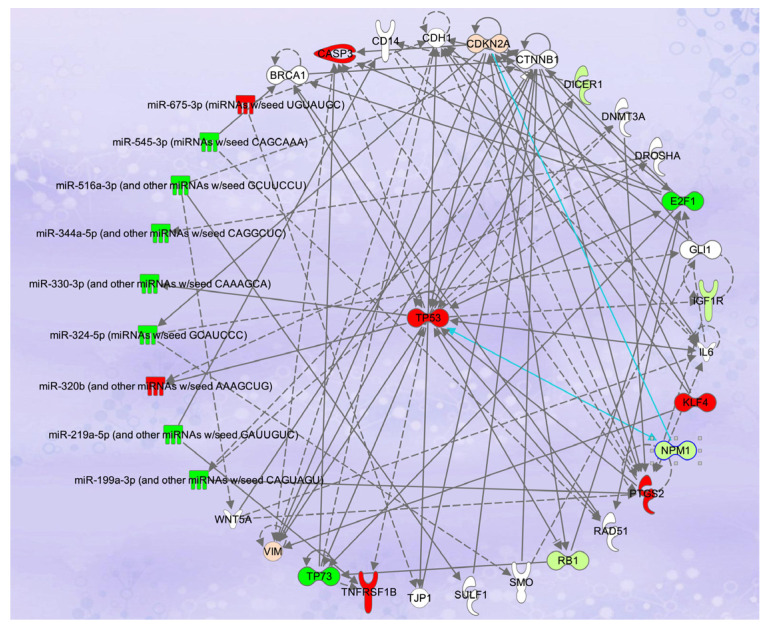
Next-generation sequencing shows associations between mRNA and miRNA expression in vitamin A and D-treated HCT-116 colon cancer cells. Apoptosis and autophagy canonical pathways were significantly upregulated, as were tumor suppressors, such as *TP53*. Upregulation of *TP53* gene expression was associated with the downregulation of numerous MiRNAs. Colorectal cancer metastasis signaling was significantly downregulated. Genes and miRNAs highlighted in red were significantly upregulated, while genes and miRNAs in green are significantly downregulated. Transcriptomic analysis showed that the expression of specific miRNAs was significantly correlated with the expression of specific mRNAs (FDR < 0.05).

Treatment of multiple cancer cell lines, including HCT-116 and SW480 colon cancer cells, with a combination of ATRA + D2 + D3 inhibited the proliferation of these cells more effectively than any of the vitamins alone [133]. The combination was highly synergistic in HCT-116 colon cancer cells and significantly reduced the IC_50_ (*p* < 0.0001), as well as time to cell death [133]. Bioinformatic analyses of the transcriptomic (RNA-seq) data showed that > 8400 genes in many interconnected canonical pathways and biological networks were differentially expressed [134]. Network pathway analysis of the data showed the involvement of multiple cancer-related canonical pathways, including apoptosis (Figure 4) and autophagy, mechanisms of cancer, histone deacetylation, as well as regulation of the epithelial–mesenchymal transition (EMT) and immunity pathways [134]. Expression of 42 genes in the EMT was altered in treated HCT-116 cells, including significant upregulation of the collapsin response mediator protein-1 (*CRMP1*) mRNA, a protein known to suppress the EMT. Furthermore, in treatment of HCT-116 colon cancer cells with the vitamin A and D combinations upregulated the expression of A Disintegrin and Metalloproteases (ADAMs), including *ADAM23* [134]. Downregulation of *ADAM23* expression in cancer cells is associated with increased proliferation, invasion, and an overall poorer prognosis. Interestingly, treatment of the HCT-116 colon cancer cells with ATRA + D2 + D3 also upregulated the communication between innate and adaptive immune cells canonical pathway, including transcripts for the toll-like receptors, as well as both interleukin-12 (*IL-12*) and *IL-15* [134]. Interleukin-12 is a naturally occurring cytokine known to regulate adaptive immunity, and it plays a primary role in interferon production, as well as the antitumor immune response [138]. Thus, these results suggest that, along with other mechanisms of action, the combination of vitamin A and D may have antitumor effects by enhancing the immune response, but further studies are needed [134]. In vivo, Crl:NU(NCr)-Foxn1nu mice with ectopic HCT-116 colon cancer xenografts and treated with vitamin A (retinyl acetate, 25,000 IU/kg) plus 25-OH-D3 (4000 IU/kg) or vitamin A (35,000 IU/kg) and vitamin D3 (5000 IU/kg) exhibited a dose-dependent reduction in tumor volume by ~38% and ~58%, respectively (*p* < 0.001) [135]. In this study, no toxicity or adverse effects were associated with treatment with high doses of these vitamins [135].

Along with mRNAs, RNA-seq also allows for parallel sequencing of multiple molecules, including microRNAs (miRNA). MicroRNAs are small, single-stranded, non-coding RNA molecules (approximately 20–25 nucleotides in size) that bind to the 3′ untranslated region of target RNAs and alter mRNA expression [139]. In colorectal cancers (CRC), miRNAs play a significant role in cancer initiation and progression, and the expression of numerous miRNAs is significantly altered [139,140]. Transcriptomic analysis (miRNA-seq) of HCT-116 colon cancer cells treated with combinations of vitamins A and D showed that multiple miRNAs associated with apoptosis were downregulated (Figure 4) [135]. The expression of these miRNAs was correlated with the expression of mRNAs in the same cell line and showed that downregulation of specific miRNAs was correlated with upregulation of the genes for the tumor suppressors *PTEN* and *TP53*, vimentin (*VIM*), and caspase 3 (Figure 4) [135]. Treatment with the vitamin A and D combination also upregulated the expression of miR103-3p, miR503-5p, miR338-3p, and let-7a-5p in HCT-116 cells. The expression of these miRNAs was correlated with downregulation of the cell cycle genes cyclin D1 (*CCND1*), cyclin E1 (*CCNE1*), and cyclin E2 (*CCNE2*). Furthermore, miR-494, a miRNA often upregulated in cancers and a promoter of metastatic disease, was significantly downregulated in vitamin A and D-treated HCT-116 cells, suggesting that this combination may reduce cancer cell invasion and metastatic disease [135]. Other researchers have reported that the antiproliferative effects of vitamin D in HCT-116 cells were associated with alterations in the expression of miR-1278, miR-627, and miR-22 [141,142]. In addition, Liu et al. [143] showed that ATRA treatment of HCT-116 cells upregulated the expression of the tumor-suppressor microRNA, miR-3666, and downregulated the proto-oncogene, E2F7, that resulted in reduced proliferation and metastasis. The increased expression of miR-3666 and decreased expression of E2F7 inhibited the activation of the MAPK/ERK signaling pathway, which plays a crucial role in the progression of cancer [143].

In breast cancer cells and ectopic MCF-7 xenograft models, vitamin A (ATRA) and D combinations were synergistic and more effective than either vitamin alone [136,137]. Combination treatment induced apoptosis in cultured MCF-7, T47D:A18, and SkBR3 breast cancer cells, with the best effect in the estrogen-positive MCF-7 cell line [136]. In terms of mechanisms of action, MCF-7 breast cancer cells treated with a vitamin A and D combination exhibited significant upregulation of genes in the unfolded protein response and apoptosis canonical pathways, and downregulation of estrogen-driven S phase entry and estrogen signaling canonical pathways, indicating that these compounds exert antiestrogenic effects as part of their mechanisms of action [136]. In addition, transcriptomic analyses of vitamin A and D-treated MCF-7 cells showed significant downregulation of genes in the epithelial–mesenchymal transition (EMT) canonical pathway, suggesting a reduction in metastatic potential of these cells. Deep sequencing further showed that multiple genes in the P13K/Akt/mTOR (PAM) signaling pathway were downregulated, including *ITBG* (integrin), *P13K*, *Akt*, *mTOR*, and *NF-κβ*, and associated with apoptosis (Figure 3) [137]. In terms of in vivo data, treatment of ectopic MCF-7 xenografts in athymic nude ovariectomized female mice with a high dose combination of vitamins A and D (25,000 IU/kg and 10,000 IU/kg, respectively) reduced tumor volume up to 70% in both a dose- and time dependent manner, with no adverse events observed after eight weeks of treatment [137]. Thus, overall, the in vitro and in vivo data suggest that the use of combinations of vitamin A and D for the treatment of breast and colon cancers may act synergistically to reduce tumor load.

**Figure 5:**
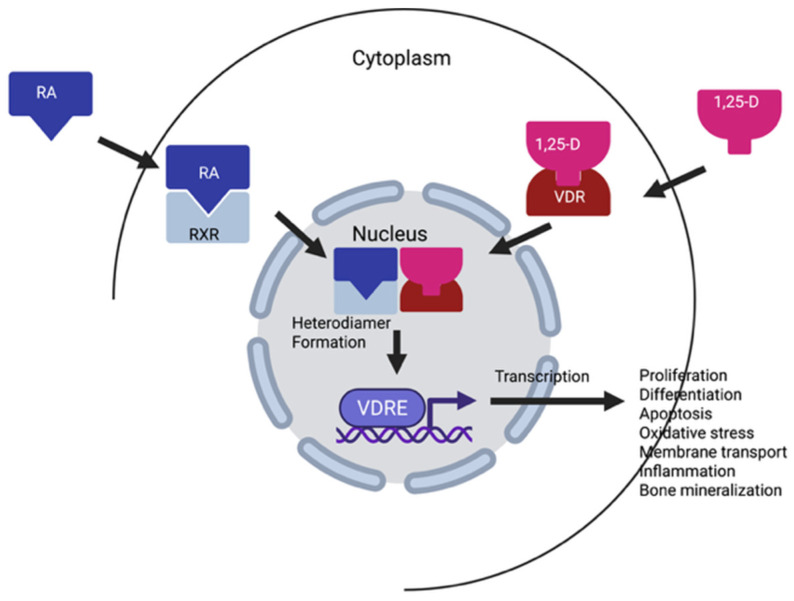
Calcitriol (1,25-OH-D) and retinoic acid (RA) bind to their respective receptors, VDR and RXR. The ligand-bound receptors form a heterodimer in the nucleus that binds to the vitamin D response element (VDRE) and initiates the transcription of genes found in many cellular processes.

Using combinations of vitamins A and D for the prevention and treatment of cancer makes sense considering the significant crosstalk between their receptors [144,145,146]. The vitamin D receptor (VDR) and retinoic acid receptors (RAR, RXR) are members of the steroid/thyroid hormone/retinoid nuclear receptor superfamily that are ligand-activated and regulate gene expression by binding to specific DNA sequences [144,145]. Ligands for both VDR (calcitriol) and RXR (retinoids and other compounds) can synergistically activate the VDR-RXR heterodimer, leading to an amplified transcriptional response. Upon ligand binding, their activation regulates the transcription of many genes associated with apoptosis, bone mineralization, cell growth, differentiation, invasion, and metastasis, and they have overlapping effects on the immune system [105,106,144,145]. The RXR is unusual in that it heterodimerizes with both the RAR or the VDR, and this heterodimerization is required to activate them (Figure 5). The binding of calcitriol (1, 25-OH-D) to the VDR initiates a conformational change in the receptor that allows it to bind to the RXR. Ligands for the vitamin A receptors include all-trans-retinoic acid, that binds to the RAR, and 9-cis-retinoic acid, that binds to both to the RAR or the RXR. Once ligand-activated, these receptors form heterodimers that translocate to the cell nucleus and bind to vitamin D response elements (VDRE) in the promoter region of target genes to regulate the expression of genes that encode for many enzymes, as well as binding and structural proteins, [144,145]. Additionally, the binding of the VDR-RXR complex to a VDRE triggers the release of corepressors and the recruitment of coactivator proteins. These coactivators work with the complex to modify chromatin (epigenetic modifications) and recruit the transcriptional machinery needed to activate or repress target genes. Since the RXR heterodimerization with the VDR is necessary for activation (Figure 5), as well as gene transcription or repression, it is logical that combinations of vitamin A and D would have synergistic effects and induce (or inhibit) the transcription of a wider range of genes in multiple signaling pathways, as well as reduce retinol and chemotherapeutic resistance. The interaction between VDR and RXR is not simple, but a complex system of crosstalk with multiple layers of regulation. The activity of the VDR-RXR complex is influenced by the cellular context, including the specific isoforms of RXR present and their relative concentrations, as well as the availability and concentration of the VDR and RXR ligands that affect both the formation and activity of the heterodimer [144,145,146].

It has also been suggested that using combinations of vitamin A with 1,25-OH-D to treat cancer, infections, autoimmune, and inflammatory diseases may reduce the adverse effects associated with high doses of vitamin D, such as hypercalcemia [144,145]. Vitamin D-related hypercalcemia has been observed in patients taking very high doses over an extended time period. Doses of vitamin D3 > 50,000 IU/day and serum levels > 150 ng/mL have been associated with hypercalcemia [144]. Short-term ingestion of vitamin D3 at a dose of 10,000 IU/d of vitamin was associated with serum concentrations of 25-OH-D below 50 ng/mL (125 nmol/L) [144].

### 2.8. Vitamins A and D Stimulate Immune Responses

Importantly, both vitamin A and D also play a significant role in the immune response [146,147,148,149,150]. VDR-RXR crosstalk significantly influences the immune system by acting as a major regulator of both innate and adaptive immunity [144,147]. The VDR-RXR complex acts on innate immune cells, including monocytes and macrophages, to modulate the initial inflammatory response to infection. The activated VDR-RXR complex, driven by vitamin D, primarily functions to suppress the production of pro-inflammatory responses and promote tolerance, which is crucial for preventing autoimmune diseases and overreactions to pathogens, such as cytokine storm seen in COVID-19 patients. Treatment of monocytes and keratinocytes with vitamin D stimulated innate immune responses by increasing VDR-dependent transcription of cathelicidin (CAMP), an antimicrobial peptide [144,147]. In one study, treatment of human monocytic leukemia cells with a 1,25-OH-D and retinoic acid combination increased the expression of the VDR proteins and CAMP, as well as induced differentiation into monocyte-macrophage-lineage cells [148]. In HCT-116 colon cancer cells, treatment with vitamin A and D combinations upregulated gene expression in the “communication between innate and adaptive immune cells canonical pathway”, including interleukin 12, a cytokine that regulates immune responses, by linking innate and adaptive immunity [134]. These data suggest that treatment with combinations of vitamin A and D may also improve the immune response in CRC.

## 3. Materials and Methods

Over the past ten years, numerous meta-analyses have focused on the impact of vitamins A and D on breast and colorectal cancer risk, incidence, progression, and mortality. For this review, we performed literature searches for studies that detailed the clinical effects of vitamins A and D on breast and colon cancer in more recent meta-analyses, clinical trials, preclinical studies, next-generation sequencing, and molecular mechanisms of action of vitamins A and D and combinations thereof.

Searches of numerous electronic databases, including the Cochrane Library, PubMed Central/Medline, Embase, Google Scholar, Napralert, and Scopus electronic databases, were performed from 15 June 2012 to 15 May 2025, without language restrictions, using relevant keywords in both free text and Medical Subject Headings (MeSH terms) format.

**Figure 6:**
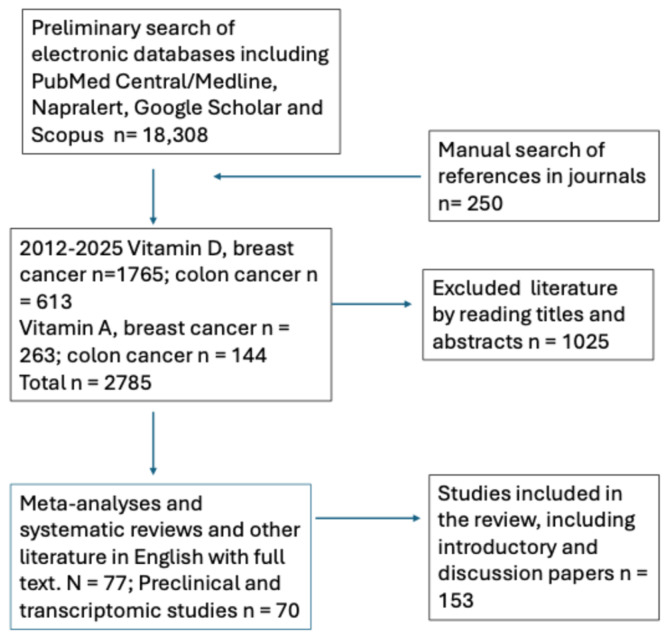
Flow diagram of the search process, including databases, number of publications (n), number of studies per category (meta-analyses, preclinical, transcriptomic), and inclusion and exclusion criteria.

Terms for the searches of the scientific and medical literature included vitamin A or D, retinoids, retinoic acid, all-trans-retinoic acid, carotenoids, cholecalciferol, calcifediol, calcitriol, breast cancer, colorectal cancer, transcriptomics, deep sequencing, next-generation sequencing, RNA-seq, meta-analysis, systematic review, and others. The Boolean search connectors used included AND, OR, and NOT, and publications in all languages were reviewed. Searches of the alternative literature were conducted in UIC repositories, catalogs (UIC) for books, abstracts, and websites (OpenGray, GetNet International), as well as conference proceedings of both national and international congresses (Figure 6).

Two researchers independently extracted data from the studies, and disagreement between them was resolved by consensus with a third researcher. The available data that correlated the effects of these vitamins on breast and colorectal cancers were analyzed. A critical analysis of these data was performed, and possible future directions for this research were suggested.

## 4. Conclusions and Future Research

Despite being amply present in meat, fish, and fortified dairy products, as well as a wide range of fruits and vegetables, the daily intake of vitamins A and D remains inadequate in a significant portion of the global population, including the USA [151,152]. Considering the inverse correlation between vitamin A and D levels and cancer, it is critical for human health to understand this relationship in the context of the increasing risk of breast, colon, and other cancers. For vitamin D, results from meta-analyses have consistently demonstrated that a vitamin D deficiency was associated with an increased risk of developing breast cancer, in both premenopausal and postmenopausal women. Furthermore, sufficient circulating 25-OH-D levels have been linked with improved survival outcomes, lower tumor grades, and fewer ER- or triple negative breast cancers in newly diagnosed patients. For women, these data highlight the importance of maintaining sufficient vitamin D levels, especially in those of African descent and those with advanced disease or obesity.

While some studies addressed vitamin D intake through diet and supplements, serum concentrations of 25-OH-D also appeared to be a significant factor in breast cancer development and outcomes. Some studies reported that daily vitamin D ingestion may also reduce cancer mortality, highlighting the importance of maintaining adequate vitamin D levels even after cancer diagnosis and during treatment. For these patients, an individualized dosing approach may be needed, with treatment with higher doses of vitamin D for short periods (50,000 IU/week for 6–12 weeks) and then lower oral maintenance doses once sufficiency is achieved. However, the daily vitamin D doses to maintain sufficiency levels may need to be titrated to the individual person, as 600–1000 IU per day does not appear to be effective in women with BC. It should be noted that serum levels of vitamins A and D should be tested on a regular basis to ensure that these higher levels are maintained.

Although the data is inconsistent for vitamin A’s effect on colorectal cancers, the results from meta-analyses show a significant inverse relationship between vitamin A ingestion and the risk and survival rates of breast, ovarian, and cervical cancers. Several studies suggested that higher dietary consumption of vitamin A, particularly in Asian and North American women, may reduce the incidence of these cancers, although the effects of supplements alone remain unclear. Inconsistencies still exist across studies, and the supporting evidence for vitamin A remains mixed. Further research, including RCTs with improved methodologies, especially for measuring vitamin A levels and the development of standardized biomarkers for vitamin A status, as well as dose-response, is urgently needed to clarify these effects for breast cancer.

Observational studies and mechanistic evidence suggested that higher serum 25-OH-D concentrations were associated with reduced CRC risk and mortality. However, the optimal dose for these antineoplastic effects remains unclear and requires further investigation in RCTs. The current evidence suggests that at least maintaining a serum 25-OH-D level in the sufficient range and potentially higher may be important for reducing cancer-specific and overall mortality. It should be pointed out that the doses needed to achieve “sufficiency” in African American and older men and women, as well as patients with obesity or cancer, may be much higher than the current daily recommendations. New RCTs are needed to investigate the anticancer effects of these vitamins in relation to a dose–response before general clinical recommendations may be made. Furthermore, studies that explore genetic polymorphisms should also be prioritized in future studies.

Investigations involving combinations of vitamins are still in their infancy but appear to be promising. In cancer cells, combinations of vitamin A and D induced apoptosis, autophagy, altered gene expression, generated reactive oxygen species, induced cell cycle arrest, and modulated multiple signaling pathways associated with mechanisms in cancer. While in vivo studies suggested that doses upwards of 25,000 IU vitamin A and 10,000 IU vitamin D may be needed to reduce tumor load, further preclinical studies are necessary to determine optimal doses, treatment periods, pharmacokinetics, and toxicity before new clinical trials of vitamin combinations are initiated.

## 5. Limitations of the Review

Randomized controlled clinical trials (RCTs) and meta-analyses make up the foundation of evidence-based medicine and are required to prove causality. While some meta-analysis for vitamins A and D included RCTs, many other studies that found a correlation between the serum levels and vitamin A or D and reductions in breast or colon cancer risks included observational and other studies that are not considered to be high-level evidence and thus may be insufficient to show causality. Randomized controlled trials (RCTs) are considered the “gold standard” for establishing causality because their design systematically eliminates the factors that can cause a correlation to be mistaken for causation. RCTs are designed to prove causality, while statistical analyses that indicate the extent to which two or more variables are associated are correlation. Only RCTs can establish causality by controlling one variable (the intervention) and randomly assigning participants to control or experimental groups, minimizing confounding variables and showing a direct cause-and-effect relationship. In contrast, correlation simply shows that two variables change together but does not prove that one causes the other, as a third, unmeasured factor (a confounding variable) may influence both. Overall, the results from RCTs have been mixed at best, due to numerous issues including poor methodology, no dose–response investigated, variations in the vitamin A and D derivatives and doses used, length of the trial study period, and the heterogeneity of breast and colorectal cancers. Furthermore, confounding factors, including alcohol consumption, diet, genetics, obesity, exercise, and smoking all are not included in all RCTs due to costs involved and feasibility. Finally, while data from pre-clinical in vitro and in vivo studies may not always translate into clinical significance due to many factors, these studies are necessary to form a foundation upon which RCTs may be based.

## Figures and Tables

**Table 1:** Key findings from analyses of vitamin A and breast cancer (BC) risk, including study limitations.

Reference	Type of Studies	Subjects	Size Effect *	Limitations	Clinical Outcomes
Eliassen et al. [40]	Cohort/case–control	3055 cases 3055 controls	α-carotene RR = 0.87 β-carotene RR = 0.83 Total carotenoids RR = 0.81	Confounded by other dietary compounds. Only one blood sample per participant.	Higher circulating carotenoids reduced BC risk.
Eliassen et al. [41]	Nested case–control	2188 cases 2188 controls Pre- and post- menopausal	α-carotene RR = 0.54 β-carotene RR = 0.32 Total carotenoids RR = 0.48	Confounded by other dietary compounds. Carotenoid measurement inconsistencies.	Higher circulating carotenoids reduced BC risk.
Sesso et al. [42]	Prospective cohort/nested case–control	508 BC cases 508 controls	Lycopene RR = 0.95	Single baseline measurements. Did not assess the long-term stability of the lycopene samples. Higher dietary lycopene was associated with lower BMI, smoking rates, reduced familial BC history, increased exercise, and fruits and vegetable intake, thus confounded.	Increasing lycopene doses did not reduce BC risk
Han et al. [46]	Cohort/case–control	25,363 BC cases, 42,281 controls	High dietary Vitamin A OR = 0.83, Dietary plus supplements OR = 0.81 High circulating vitamin A OR = 1.0	Different ethnicities, Publication biases, language issues, and vitamin A measurement differences.	Higher dietary intake and supplementation of vitamin A lowered the incidence of BC in North American and Asian women, but not women from Oceania or Europe
Hu et al. [47]	Nested case/case–control	NS Pre- and post-menopausal	Highest plasma retinol versus lowest OR = 0.81 Plasma retinol OR = NS	Possible measurement issues, unmeasured or residual confounders, moderate to high heterogenicity in analysis. Lack of original data.	No significant association between plasma retinol and vitamin A levels and BC was observed.
He et al. [48]	Epidemiological studies	19,450 Pre-diagnosis 2990 BC cases, 502 deaths; post-diagnosis 16, 460 BC cases, 1823 deaths	Pre-diagnosis α-carotene HR = 0.9 β-carotene HR = 0.7 Post-diagnosis HR 0.92–1.17	Misclassification of vitamin A intake. The food questionnaire used introduced measurement errors. Heterogenicity in pre-versus post-diagnosis intakes, variation in study methods, no adjustment for tumor stages, and few studies adjusted for BMI, physical activities, or healthy lifestyles.	Higher dietary ingestion of β-carotene increased breast cancer survival by 30%, but other vitamin A derivatives, including α-carotene, β-cryptoxanthin, lycopene, retinol, or lutein, had no effect.
Li et al. [49]	Cohort	17,062	HR = 0.92	Multiple vitamins were used, including vitamin A; small sample sizes, heterogenicity in study designs; different countries and lifestyles; no staging of BC; descriptions of vitamins were lacking.	No significant difference in vitamin A or E use in breast cancer survival, and only vitamin C intake after breast cancer diagnosis was significantly associated with better overall survival
Kim et al. [50]	150 Cohort/nested case–control studies; randomized clinical trials	NS	NS	Some of the studies included had poor design and protocols; variations in the vitamin A derivative used and doses; variations in the length of the trial study period, and the heterogeneity of breast cancer; the methodology for measuring serum levels of vitamin A was inconsistent.	Inverse relationship between concentrations of retinoids and carotenoids and the risk of BC and premalignant breast disease; Inverse relationship between incidence, recurrence, and survival of aggressive tumors

* RR = Relative Risk; OR = Odds Risk; HR = Hazard Ratio are three common statistical measures used in clinical research. RR (Relative Risk) is the ratio of the probability of an event in an exposed group versus an unexposed group, representing a cumulative risk over the study’s duration. OR (Odds Ratio) compares the odds of an event occurring between two groups and is often used in case–control studies. HR (Hazard Ratio) compares the rate of an event occurring over time between two groups, representing an instantaneous risk.

**Table 2:** Key findings from analyses of vitamin D and breast cancer (BC) risk, including study limitations.

Reference	Type of Studies	Subjects	Size Effect	Limitations	Clinical Outcomes
Este Estébanez et al. [56]	Cohort (CO)/case–control (CC)	Not reported	CO RR = 0.85 CC RR = 0.65 For the relationship between vitamin D and breast cancer. for premenopausal women only RR = 0.67, no effect for menopausal women	Variability in the literature used. Different cutoffs for vitamin D levels in studies. No dose response.	Analysis showed a protective effect between higher serum levels of 25 (OH) D and BC in both cohort studies and case–control studies. But when menopausal status was included, the protective effect of vitamin D was only significant for premenopausal women if the analysis was restricted to nested case-control studies. No conclusions could be made for vitamin D intake or supplements.
Hossain et al. [57]	Case–control/nested case–control	229,597	Vitamin D deficiency RR = 1.91 Serum vitamin D levels vs. BC occurrence RR = 0.99 Vitamin D intake RR = 0.99 Vitamin D supplements RR = 0.97	Studies used were restricted to three public databases; no cross-referencing was performed; low-powered studies used; evidence from observational studies included.	Vitamin D deficiency is directly related to BC occurrence. No significant effect found for vitamin D intake or 1,25-OH-D2.
Yao et al. [58]	Cohort	3995 women with BC Stratified for 25-OH-D levels <20 ng/mL deficient; 20–30 ng/mL insufficient; ≥30 ng/mL sufficient	Survival Sufficient at diagnosis HR = 0.73; BC specific HR = 0.78; recurrence free survival HR = 0.79	Different ethnicities; publication biases; and different measurement methods	African American women with BC had the lowest serum 25-OH-D levels, as well as the worst prognosis and poorer survival. The analysis showed a protective effect between higher serum levels of 25-OH-D and BC, versus lower levels of 25-OH-D.
Rosso et al. [59]	Cohort	292 pre- and post-menopausal women with BC	RR = Not reported	Single-center study; variations in BMI; co-morbidities were not included in analysis; lack of healthy controls; vitamin D levels only measured at time of diagnosis.	Approximately 65% of newly diagnosed women with BC were vitamin D deficient, including 56% of younger women. Vitamin D deficiency in newly diagnosed patients is associated with higher tumor grade and advanced stage BC.
Mackey et al. [60]	Cohort, women over 40 yrs of age	Stratified for vitamin D levels <20 ng/mL 73,659 ≥30 ng/mL 73,659	<20 ng/mL RR = 1.45 postmenopausal RR = 1.18 pre-menopausal	Possible measurement issues, unmeasured or residual confounders, moderate to high heterogenicity in analysis.	Low vitamin D levels (≤20 ng/mL) in postmenopausal women were associated with a significant 45% increase in the risk of developing BC. Pre-menopausal women with deficient vitamin D levels had a significant 18% increased risk.
Ottaiano et al. [61]	Cohort	722 for NACT response; 1033 for PFS; Stratified for low/deficient vitamin D levels versus high/sufficient vitamin D levels	NACT high levels OR = 0.78 High vs. low for disease progression HR = 0.65	Variation in study heterogenicity and methods; no adjustment for tumor stages; limited vitamin D assessments.	Adequate baseline 25-OH-D levels are associated with a significant 22% reduction in the risk of a non-response to Neoadjuvant Chemotherapy and a significant 35% reduction in the risk of disease progression.

**Table 3:** Key findings from analyses of vitamin D and CRC risk, progression and mortality, including study limitations.

Reference	Type of Studies	Subjects	Size Effect	Study Limitations	Clinical Outcomes
Hernández-Alonso et al. [72]	Cohort (CC)/case–control (CO)	140,112	OR = 0.61 in CC OR = 0.8 in CO	Variability in the international literature used, and no lifestyle issues were addressed. Only two public databases were used. Different methods and cutoffs for vitamin D analyses and levels; No dose response.	A 39% risk reduction in CRC was found in the highest vs. the lowest levels of total 25(OH)D; a 20% reduced CRC risk was seen in prospective cohort studies. Results in women were significant, while results in men were non-significant
Huang et al. [75]	Case–control/nested case–control	>800,000	Women RR = 0.63 Men RR = 0.89 Asian populations RR = 0.67 European and USA RR = 0.82 Vitamin D intake and CRC risk RR = 0.81 High vitamin D overall survival outcomes HR = 0.69 CRC survival RR = 0.64 Left-sided CRC RR = 0.6	Studies used were restricted to three public databases; no cross-referencing.	High circulating 25-OH-D reduced CRC risk. Vitamin D and calcium have an additive effect on CRC incidence, transformation, and progression, especially for women and left-sided CRC patients
Ottaiano et al. [76]	Randomized controlled clinical trials (RCTs)	>1800	Lower 25-OH-D Risk of death HR = 1.47 Risk of progression HR = 1.38	Different analytical methods and cutoffs for vitamin D levels and vitamin D concentrations were expressed using different measurement units. Vitamin D level cutoff values (low vs. high) also varied. Chemotherapy regimens were not discussed, nor were prognostic factors such as the initial disease burden, treatment responses, and toxicity.	Low vitamin D levels increased the overall risk of disease progression and mortality in metastatic CRC patients
Keum et al. [77]	RCTs	6537	CRC incidence RR = 0.98 Mortality RR = 0.87 with daily dosing	Included RCTs were not designed to test the hypothesis that vitamin D influenced the risk of cancer incidence or mortality. Over sampling of Caucasians versus other ethnic groups. Lack of data on site-specific cancers.	A significant 13% decrease in cancer mortality and 7% reduction in total mortality over 3–10 years of follow-up, which was attributed to daily and not bolus dosing of vitamin D.
Arayici et al. [78]	Meta-analyses of RCTs, observational, and epidemiological studies	>1,000,000	Higher 25-OH-D levels associated with reduced cancer risk OR = 0.93 Cancer mortality OR = 0.67 Vitamin D intake and CRC-specific risk OR = 0.89 Vitamin D intake and overall cancer mortality OR = 0.89	A large portion of the studies included in the research (77.1%) were also observational studies. The primary endpoints of many studies included in the meta-analyses of RCTs were not focused on cancer incidence or cancer-related deaths. RCTs did not measure vitamin D levels at the conclusion of the studies. Differences in dosing protocols between studies.	Higher Vit-D intake and serum 25(OH)D levels were associated with lower cancer risk and cancer-related mortality. In subgroup analysis, Vit-D intake was associated with a significant decrease in CRC incidence.
Zhang et al. [79]	RCTs	72,669	RR = 0.96	A limited number of databases were used for the study. Limited number of studies and sample sizes.	Vitamin D supplementation did not reduce overall cancer mortality.
Guo et al. [80]	RCTs	60,876 Vitamin D intervention; 60,653 controls	RR = 0.98 Vit D supplementation mortality RR = 0.88	Due to the wide variations in vitamin D treatments and dosing, the analysis could not effectively assess the equivalent daily dose of vitamin D supplementation. Total cancer incidence or mortality were secondary outcomes of the included RCTs. Not all RCTs reported associations between different doses of vitamin D intake and total cancer incidence and mortality. While RCTs reported country information, the longitude and latitude were not reported. This may be a confounder, as it is well known that people in lower latitudes tend to have higher 25-OH-D levels.	Pooled data from RCTs showed that vitamin D supplementation, with/without calcium, did not reduce total cancer incidence of breast or colon cancers. However, vitamin D supplementation significantly reduced total cancer mortality by 10%. Total cancer mortality was observed when 25-OH-D levels were <40 ng/mL and the baseline mean < 20 ng/mL.

**Table 4:** The Scientific Advisory Committee on Nutrition (SACN) in the UK has recommendations for vitamin D levels and status (www.gov.uk).

Serum 25-OH-D ng/mL	Vitamin D Threshold and Treatment Advice
<30	Deficient-treat
30–50	Sufficient for most healthy people. Treatment is advised for patients with fragility fracture, osteoporosis, or high fracture risk, patients taking antiresorptive drug therapy, symptoms of vitamin D deficiency, reduced exposure to sunlight, darker skin tones, religious/cultural dress, and for those patients taking parathyroid medications, antiepileptic drugs, oral glucocorticoids, or malabsorption syndrome.
>50	Sufficient for the whole population, but levels need to be maintained by dietary supplementation or safe sunlight exposure

**Table 5:** Doses and serum levels of vitamin D associated with clinical trials and outcomes.

Reference	Vitamin D Dose	Serum Level	Clinical Outcomes
Giovannucci et al. [84]	Each 100 IU D3 raised the serum level of 25-OH-D by 1.75 nmol/L (0.7 ng/mL)	Increase of 25 nmol/L	17% reduction in total cancer in men
			29% reduction in total cancer mortality in men
			43% reduction in GI cancers in men 45% reduction in GI cancer mortality
Crew et al. [85]	400 IU/day D3 for one year	Increased serum levels of 25-OH-D by less than 3 ng/mL over one year	Administration of 400 IU/day D3 did not increase 25-OH-D levels into the sufficient range in women with breast cancer taking zoledronate
Khan et al. [86]	50,000 IU/week D3 for 12 weeks, then 600–1000 IU daily for 3–6 months	Increased serum 25-OH-D to 66 ng/mL	High-dose vitamin D3 increased the 25-OH-D levels into the sufficient range in breast cancer patients taking letrozole. But maintenance doses of 600–1000 IU/day were not high enough, and the levels dropped by 7%/month
Peppone et al. [87]	Low dose: 1000 IU/day High dose: 50,000 IU/week D3	Increased serum 25-OH-D by 24.3 ng/mL after 8–16 weeks	High-dose vitamin D3 increased the 25-OH-D levels into the sufficient range in women with BC, while the low dose did not

**Table 6:** Doses, clinical outcomes, and adverse events associated with vitamin A clinical trials.

Reference	Vitamin A Dose	Serum Level	Clinical Outcome
Alberts et al. [90]	50,000 to 75,000 IU/day for 12 months	Not tested	Reduced squamous cell carcinoma. High doses of vitamin A were safe in patients with severe sun damage. No toxicity observed
Moon et al. [91]	25,000 IU/day	Not tested	Reduced squamous cell carcinoma but had no effect on basal cell carcinoma. No adverse effects reported.
Cartmel et al. [92]	25,000 IU/day for 3.8 years	Not tested	High-dose vitamin A intake for an extended period of time was associated with an increased alkaline phosphatase level (7%); higher triacylglycerols (11%), higher cholesterol (3%), and a lower HDL (1%) in the retinol group than in the placebo group
Goodman et al. [93]	Up to 200,000 U/m^2^ (~350,000 IU presuming a 150-pound person)	Not tested	High dose vitamin A increased triglycerides, increased headaches and emotional instability, mild skin and mucous membrane dryness, and, in some cases of hepatomegaly associated with vitamin A toxicity

**Table 7:** National Institutes of Health/National Academy of Medicine vitamin D recommendations. (www.nam.edu).

Life Stage	Recommended Amount
Birth–12 months	10 mcg (400 IU)
Children 1–13 years	15 mcg (600 IU)
Teens 14–18 years	15 mcg (600 IU)
Adults 19–70 years	15 mcg (600 IU)
Adults > 70 years	20 mcg (800 IU)
Pregnant and breastfeeding women	15 mcg (600 IU)

**Table 8:** Summary of the transcripts altered in breast cancer cells treated with vitamins A or D.

Cell Line Tested and Vitamin Treatment	Transcripts Altered
Breast, colon, vitamin D [124]	Oncogenes: *MYC*, *JUN*, *AP-1*, *JUNB*, *JUND*, *FOS* Tumor suppressor genes: *CCNC* (cyclin C), *CCND1*, *CDKN1A*, *CDKN1B* and *G0S2* (G0/G1 switch 2)
MDA-MB-231 and MDA-MB-468, all-trans-retinoic acid (ATRA) [125]	ATRA-regulated genes: keratin 7 (*KRT7*), prostaglandin E synthase (*PTGES*), dehydrogenase reductase 3 (*DHRS3*), nuclear receptor interaction protein 1 (*NRIP1*), and cytochrome p450 family 26A1 (*CYP26A1*)52. Transcription factors: interferon regulatory factor 1 (*IRF1*) and myocyte enhancer factor 2 (*MEF2*)
MCF7 cell line, and a RA-resistant BT474 cell lines, ATRA [126]	STE20-like protein kinase 4 in MCF7 cells, Ppp4r3a protein phosphatase 4 regulatory subunit 3A (*SMEK1*) in BT474 cells; Transforming growth factor-1beta (*TGF1β*) in MCF-7 cells, bone morphogenetic protein and activin membrane-bound inhibitor (*BAMBI*) in BT474

## Data Availability

The data used to generate this review were obtained from public domain resources. Thus, all the data used in the generation of this review are freely publicly available.

## Abbreviations

The following abbreviations are used in this manuscript:

A Disintegrin and Metalloproteases	ADAMs
All-trans-retinoic acid	ATRA
Breast cancer	BC
Bone morphogenetic protein and activin membrane-bound inhibitor	BAMBI
Calciferol	25-OH-D
Calcitriol	1,25-OH-D
Cathelicidin	CAMP
Cholecalciferol	vitamin D3
Collapsin response mediator protein-1	CRMP1
Colorectal cancer	CRC
Cyclin D1	CCND1
Cyclin E1	CCNE1
Cyclin E2	CCNE2
Epithelial–mesenchymal transition	EMT
Ergocalciferol	vitamin D2
Estrogen receptor	ER
Hormone responsive	HR
Inducible nitric oxide synthase	iNOS
Interleukin-12	IL-12
Interleukin 15	IL-15
Integrin	ITBG
Mammalian target of rapamycin	mTOR
Non-adjuvant chemotherapy	NAC
Nuclear Factor kappa beta	NF-κβ
Phosphoinositide 3-kinase	P13K
Protein kinase RNA-like endoplasmic reticulum kinase	PERK
Randomized controlled clinical trial	RCT
Reactive oxygen species	ROS
Retinaldehyde dehydrogenase 1A3	ALDH1A3
Retinoid receptor	RXR, RAR
Reverse Transcription polymerase chain reaction	RT-PCR
Transforming growth factor-1beta	TGF1β
Vitamin D binding protein	VDBP
Vitamin D receptor	VDR
Vitamin D response elements	VDREs
